# Current opinions: Zeros in host–parasite food webs: Are they real?^☆^

**DOI:** 10.1016/j.ijppaw.2013.08.001

**Published:** 2013-08-17

**Authors:** Wayne Rossiter

**Affiliations:** Department of Biology, Waynesburg University, 51 W. College St., Waynesburg, PA 15320, United States

**Keywords:** Food web, Parasitism, Diversity, Life cycle, Community

## Abstract

As the data have poured in, and the number of published food webs containing parasites has increased, questions have been raised as to why free-living species consistently outnumber parasites, even though most general reviews on the subject of host:parasite species richness suggest the contrary. Here, I describe this pattern as it exists in the literature, posit both real and artifactual sources of these findings, and suggest ways that we might interpret existing parasite-inclusive food webs. In large part, the reporting of free-living species devoid of any associated parasites (termed here in the coding of food web matrices as “zeros”) is a consequence of either sampling issues or the intent of the study. However, there are also several powerful explanatory features that validate real cases of this phenomenon. Some hosts appear to authentically lack parasitism in portions of their geographic ranges, and parasites are often lost from systems that are either in early phases of community re-colonization or are compromised by environmental perturbation. Additionally, multi-stage parasite life cycles and broad host spectra allow some parasite species to partially saturate systems without providing a corresponding increase in parasite species richness, leading to low parasite species richness values relative to the free-living community. On the whole, the existing published food webs are sufficient to, at least in principle, determine basic patterns and pathways associated with parasite establishment and persistence in free-living communities because (1) for the purpose of those features, species rarity is roughly analogous to absence and (2) the existing data seem to suggest that the addition of more parasite taxa would reinforce the patterns already observed. This is particularly true for helminth parasites, in which our understanding and the resolution of our work is most robust.

## Highlights

• Most free-living species are hosts for multiple parasite species.
• Food webs containing parasites display fewer parasites than free-living species.
• This pattern is largely a product of researcher intent or methodological artifact.
• However, there are also verifiable sources for this pattern, related to the nature of the system being investigated.
• While most food webs underestimate the number of parasites, the observed patterns of parasitism are likely valid.

## Graphical abstract

**Figure:**
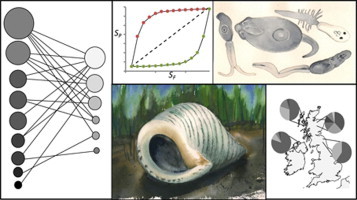

## 1 Introduction

At a recent meeting of the American Society of Parasitologists (Anchorage, AK, 2011), an important, though largely ignored, question was raised at the conclusion of my oral paper discussing the patterns of parasitism in a riverine food web; where are all the parasites? Specifically, the discussion centered around the apparent paucity of parasites in published food webs relative to free-living taxa, and spawned a recent review offered by my mentor and co-author on the paper (Sukhdeo, 2012). While the review was successful in framing the relationship between ecologists studying food webs and those of us attempting to use food webs to explain basic patterns of parasitism (and the concerns and issues therein), little attention was given to explaining why food webs typically contain far fewer parasites than free-living species. From the outset, most food webs containing parasites match (or surpass) the resolution of those containing only free-living species, and are often studies that augment (and build from) already well-established food webs. But, as a demonstration of the problem, consider a laundry list of recent parasite-to-free-living ratios in published food webs (note, I have removed micropredators as parasites where applicable); 25:88 (Huxham et al., 1995), 11:62, 40:83 (Lafferty et al., 2006a), 16:122 (Rossiter and Sukhdeo, 2011), 13:37 (Amundsen et al., 2009), 35:161 (Zander et al., 2011), 15:48 (Preston et al., 2012) and 21:100 (Rossiter and Sukhdeo, in review).

At best, we see a 1:2 ratio of parasite-to-free-living taxa, and this sits in stark contrast to the case that parasitologists have been making to the larger scientific community (that parasites are more abundant than free-living taxa and are crucially important; e.g. Poulin, 1997; Dobson et al., 2008; Lafferty et al., 2008). For example, it is frequently stated that parasitism is the most common and abundant feeding strategy in nature (Price, 1980). Parasites are present in nearly every animal group, and can make up as much as 75% of the interactions observed in biological systems (Lafferty et al., 2006b). It is now thought that we are losing many species of parasites with the extinction of each free-living species (Dunn et al., 2009). Those working with particular host–parasite systems will no doubt be able to ramble off numerous parasites associated with their favorite host species. For example, work on mummichogs (*Fundulus heteroclitus*) along two rivers in New Brunswick, Canada, produced more than 20 associated parasite species (Blanar et al., 2011). In a recent survey of the ectoparasites of 45 species of marine fishes, only one had fewer than three associated parasite species, with an average of 5.36, suggesting that parasites should be much more prevalent in our community-wide studies (Gotelli and Rhode, 2002). The same pattern emerges when considering the parasites of the gastrointestinal tracts of marine fishes (Poulin and Luque, 2003). An earlier large-scale review of parasitism across vertebrate taxa yielded an average of 10.53 (±8.39) parasite species per host (Poulin, 1997). Taken as a whole, it is estimated that there may be as many as 300,000 parasite species residing in just the 57,000 vertebrate taxa on the planet, and while the full culmination of the existing data suggest just 1.53 helminth taxa per host species (neglecting all protozoans, bacteria and viruses; Dobson et al., 2008), we still get the idea that there are many more parasites than free-living taxa in natural communities.

Given their apparent ubiquity, it is odd that every published food web containing parasites also contains many free-living species that do not act as a host for any parasites. Here, I refer to these as “zeros,” in that the coding of non-interactions in food web matrices is typically “0”. But, are these zeros real? If so, why do they exist? If not, what biases give rise to this phenomenon? I argue that nearly all zeros are artifacts of sampling biases (both unavoidable and intentional). However, some well-known patterns help make sense of real absence data in many systems, and there is some anecdotal evidence that some zeros are completely authentic. These sources of absence data in parasitological studies are summarized in Table 1. I then address how this might impact our interpretation of the existing literature in this area, and offer suggestions for future work.

## 2 Sources of zeros

### 2.1 Taxonomic resolution and sampling effort

For anyone working in the field of ecology (or parasitology for that matter), it would be hard to miss the fact that food webs have become a (if not the) prominent way for biologists to study the structure and function of natural communities. However, assembling reliable measures on entire ecological communities is an incredibly daunting task (Brown and Gillooly, 2003; Woodward et al., 2005). This is also the case for those attempting to incorporate parasites into food webs. Where studies that hone in on a particular host species might discover many associated parasites, the sampling necessary to flesh out such high-resolution data is almost impossible at the community level. Beyond the simple logistics of sufficiently sampling a system and the shear manpower necessary, whole-community studies typically rely on collaborative efforts among many biologists, each specialized in their respective floral or faunal groups. For this reason some groups are more resolved than others in food web studies. As an anecdotal example, almost no authors even attempt to resolve plankton in their systems (unless they are explicitly performing a study centered around planktonic species), instead simply calling large groups of organisms “phytoplankton” or “zooplankton,” and occasionally explicitly naming relevant ostracods or copepods (see references herein). In a recent study of the Raritan River food web in central New Jersey, I incorporated “morphospecies” of phytoplankton and zooplankton, which resulted in 12 additional organismal units in the lower trophic levels of the food web (Rossiter and Sukhdeo, in review). Still, a detailed review of 67 river systems revealed an average phytoplankton species richness of 126 (Rojo et al., 1994), suggesting that a more resolved plankton survey would more than double the size of most food aquatic food webs! The same patterns exist for microbes, unionids, and other animal groups in these systems. Likewise, it is this absence of resolution (and appropriately skilled specialists) that initially led to the lack of parasites in early food webs, the absence of which was addressed by Marcogliese and Cone (1997) among others. This situation has been fully acknowledged and evaluated in the literature (e.g. Paine, 1988; Martinez, 1991, 1993), with the general feeling being that having poorly resolved guilds or groups can impact some, though certainly not all, of the general patterns observed (Thompson and Townsend, 2000; Dunne et al., 2002; Woodward et al., 2005).

Another related and entirely unavoidable problem is the bias of sampling effort among organismal groups (taxonomic, trophically defined, or otherwise). One reason is that some habitat types are more amenable to sampling than others. For example, even at small spatial scales in lotic systems, deep and fast moving waters represent a different set of difficulties than do slower moving pools. Runs are difficult to traverse, even harder to sample benthos in (when deep), and are almost always undersampled when trapping or electroshock methods are being used. Ironically, many of us in the field have focused on wetland and marsh systems, expanses of which are nearly impossible to access, let alone sample. Likewise, sampling the epipelagic portions of seas or large lakes is expensive, labor intensive and seasonally inclement. While I’m emphasizing aquatic examples, the basic point is that some habitats or locations represent low hanging fruit that can be sampled easily and repeatedly, while others are much more difficult to study. This has serious implications where, as in the riverine example above, communities (and therefore food webs) exist across heterogeneous habitats. By the very nature of those differences, some organisms will be easier to sample than others. Because most parasitological surveys require the destructive sampling of hosts, and many parasites are not prevalent in host populations (i.e. only a small fraction of individuals are infected), hard-to-sample organisms are more likely to produce zeros that are artifacts of sampling bias.

Beyond the differences in the ease of sampling across (or within) systems, particular groups of organisms are easier, more expedient and less expensive to survey than others. Numerically, some taxa are rare while others are common. Consider Rossiter and Sukhdeo (2012), where two larval trematodes using the eastern mudsnail (*Ilyanassa obsoleta*) as a first intermediate host represented 88.6% of all infections in this snail. However, the destructive sampling of more than 5000 snails yielded only six infections of the trematode *Lepocreadium setiferoides*. In Hernandez and Sukhdeo (2008), the authors surveyed more than 4000 copepods, and never observed the larval stage of a gnathostomid nematode which had been verified in fish collected from a Pine Barrens stream (personal communication). Abundant taxa are abundantly sampled, while the parasite fauna of rarely seen species remain poorly resolved. This bias can also be related to size differences. For example, the natural abundance of benthic invertebrate fauna (say for example ephemeropterans or pleurocerids) results in the easy collection of many more specimens for destructive sampling than some larger and less abundant organism (say, Great blue heron, *Ardea herodias*). These issues (both among free-living and parasitic groups) have been extensively dealt with in the literature, and the problem of sampling bias is well known (e.g. Courchamp et al., 2000; Lenat and Resh, 2001; Pawar, 2003; Ellison and Agrawal, 2005). Where sampling numbers are insufficient to conclusively demonstrate the absence of parasites, we tend to tentatively encode these as genuine absences in our food webs, until proven otherwise. While it is highly likely that some of these free-living taxa do have one or more associated parasites, it is prudent to assume a zero, until otherwise demonstrated.

Many of the previously mentioned sources of zeros are outside of the researcher’s control. However, some sampling biases are intentional. In many studies, the absence of parasites in the free-living taxa of our food webs is not only well-known, but is directly acknowledged as a consequence of the purpose or intent of the research. For example, almost without exception, the “parasites” in food webs are better defined as “parasitic helminths” or “macroparasite” fauna, and most authors are very clear that they were not attempting to survey microparasite (viral, bacterial and protozoan) diversity. That is, the authors are explicit about what parasite groups they are looking for, and which they are ignoring. While an ornithologist studying a particular species may be fully aware of the vector-born parasitic diseases present in a particular population or locality, few community-level studies would even look for blood-borne diseases in those species. To my knowledge, none of the food webs in the existing literature have actually attempted to include such parasites in their surveys (perhaps with exception of terrestrial studies that use community-level information to understand specific viral, bacterial or protozoan parasites; see Johnson et al., 2013 for a non-food web community study).

### 2.2 Spatial and temporal scales

One of the more significant considerations in dealing with zeros in our food webs is the fact that our understanding of parasite-host complexes is often cumulative with respect to the distribution of a host species. For example, a large-scale survey of the parasites of the three-spine stickleback (*Gasterosteus aculeatus*) revealed that there were 86 parasite species associated with the fish in Eurasia alone (Poulin et al., 2011). However, site-specific data in this study displayed a different pattern at smaller scales, with many stickleback populations having only one recorded parasite species. Thus, it seems clear that spatial scale is of major consequence in dealing with absences of parasites in food webs. Simply opening a copy of the Parasites of North American Freshwater Fishes (Hoffman,1999) or searching the Fishpest database (Strona and Lafferty, 2012) will reveal that each fish has dozens of associated parasite taxa (and again, only considering macroparasites), but no one would expect to find many of these parasites in the fishes being collected at their local sampling sites. It is well known that species with large geographical distributions act as hosts for larger numbers of parasites (Gregory, 1990; Poulin, 2007), and this pattern has been related to the species-area curves of habitats for free-living taxa (because hosts *are* habitat for parasites). It follows that a host appearing in one location will likely have parasite assemblages that differ in number and identity to hosts collected from other locations (e.g. Soldánová et al., 2010). In some cases, a species known to harbor parasites will be devoid of them in particular locations. Again using anecdotal evidences (because frankly, few have thought to publish such negative findings), extensive surveys of the parasite communities of *Littoraria irrorata* along the mid-Atlantic coastline of North America, have yielded no helminth parasites (Rossiter, unpublished data, and personal communication with Brian Silliman), even though the species is known to harbor them in the southern portion of its distribution (Reid, 1984). I have observed the same phenomenon in the coffee bean snail (*Melampus bidentatum*) throughout the southern shores of New Jersey. While more nominal in its explanatory power, cases of free-living taxa being freed from any associated parasites exist in the area of invasive species biology, where invasive species are often able to escape not just predatory pressures, but parasitism too (e.g. Dove, 2000; Torchin et al., 2002).

It also seems evident that the zeros observed for some systems are a consequence of the condition or biology of the system itself. For example, Hernandez and colleagues (2007) worked in a highly acidic blackwater system, which is naturally species poor. Not only were the free-living communities depauperate, but the requisite hosts associated with many parasite life cycles were absent. So, while most of the observed free-living taxa act as hosts for many reported parasites in more traditional freshwater environments, the acrid conditions of these waters prevent successful colonization of the species necessary to maintain many parasite life cycles. While not intended to be a comprehensive survey of the system’s parasites, Anderson and Sukhdeo (2012) surveyed habitats associated with the Meadowlands saltmarsh complex, in which sites existed at different stages of recovery. As anticipated, the parasite communities of the most recently reconstructed marshes were scant, with only direct-life cycle parasites being present. Assessment of the parasite communities along a gradient of human impacts on the Raritan River has revealed that increasing perturbation leads to a reduction of the total parasite species richness of the system, demonstrating that pristine systems can harbor more parasites (Rossiter, 2012). Thus, the condition of a system (in terms of development and disturbance) can have a significant impact on local parasite communities.

It must also be remembered that, while they are not usually discussed in this context, temporal scales and survey time intervals can also explain some of the zeros in our food webs. Some studies sample relatively short segments of time (on the order of 1–3 years, or even particular seasons; e.g. Wilbur, 1997), while others represent continual collections over many years, or even decades (e.g. Emmerson and Raffaelli, 2004; Amundsen et al., 2009). Given that the presence or absence of a species in a given locality can often be described by a “blinking lights” scenario where local establishment and extirpation can occur at seasonal or yearly intervals (discussed in Morin, 2011, pg 252), studies conducted over shorter time frames may fail to capture the full diversity of parasites present in a system.

### 2.3 Animal ecology and life history

There is a way by which free-living species might outnumber parasites in food webs even when many of them harbor one or more parasites. A single parasite with a complex life cycle (having two or more requisite hosts for development and maturity) can show up multiple times in our food webs. Just as a particular invertebrate may be recorded as a host for some parasitic helminth, so too are any species that act as hosts for later larval or adult stages. Thus, a number of free-living species can be identified as hosts for the same parasite species. This is tightly related to host spectrum (the number of hosts a parasite can utilize at any given life stage). It is well known that some parasites are capable of infecting a broad range of hosts, while others are stringently constrained to particular hosts (Poulin, 2010). Generally, it has been argued that there is a cost to maintaining compatibility with many hosts, and that this acts as a selective pressure to keep the number of competent host species relatively small at each stage of the life cycle (Combes, 2001, pgs 45–94), and this has been demonstrated for a number of parasite taxa (e.g. Dick and Patterson, 2007). Prevailing thought is (and empirical work suggests) that parasite genotypes are under stringent purifying selection, producing generations (or populations) that are locally adapted to particular host genotypes (e.g. Lively and Dybdahl, 2000). This constant pressure to “chase the tail” of the most common host genotypes in a population is drastically amplified when trying to track and react to multiple hosts in a complex life cycle, or taxonomically diverse hosts at any particular stage of the life cycle (e.g. Loot et al., 2006). However, some parasites are quite generalist in their predilections for hosts, and are capable of infecting a large number of species. Additionally, the ability to “infect” paratenic hosts can be important for diversification and speciation in some systems (Marcogliese, 2002, 2007). For example, the trematode *Posthodiplostomum minimum* is a nearly ubiquitous parasite of freshwater fishes, where it encysts as a larval metacercaria. While it is suspected that this parasite actually represents a complex of cryptic species, it has been recorded in more than 100 North American fish species (Hoffman, 1999). Likewise, the adult stage has been found in more than two dozen vertebrate taxa (Palmieri, 1973, 1976). Similar cases can be made for cosmopolitan species like *Diplostomum spathecum* (163 known fish hosts), *Anisakis* spp. (256 known fish hosts) and *Contracum* spp. nematodes (131 known fish hosts, but see the issue of cryptic species in Locke et al., 2010). As demonstrated in the food web matrix used by Lafferty et al. (2006a), it is not uncommon to see more than a dozen free-living species harbor the same parasite in a system. The consequence of this phenomenon is that a single parasite can be recorded in many different hosts, meaning that, at least in principle, the number of parasites in a food web could be small, even when all free-living animals serve as hosts.

### 2.4 Trophic position

Thus far, I have tried to demonstrate that geographical constraints on parasite communities, temporal considerations, and overlaps in host spectra suggest that we might anticipate fewer parasites (relative to free-living taxa) in our food webs than previously thought. A final source zeros is a consequence of the trophic structure of food webs themselves. While gastropods may be the host of choice for most larval trematodes, free-living species that exist on lower trophic levels typically have few parasites associated with them (e.g. Chen et al., 2008; Rossiter and Sukhdeo, 2011). Thus far, this particular pattern appears nearly to be universal (but see Amundsen et al., 2009). For every copepod, ostracod or amphipod carrying a larval parasite, numerous free-living herbivores or lower omnivores are devoid of parasites in our surveys. While the bulk of parasites recorded in food webs tend to be helminthes with complex life cycles that often involve trophic transmission, there are other biological explanations that help validate this pattern. When evaluating large meta-analyses, larger or older organisms tend to carry more parasites (both in terms of richness and abundance; e.g. Lindenfors et al. 2007). But, animal size and longevity are also strongly correlated with trophic level (Romanuk et al., 2011), meaning that larger and more long-lived organisms have both more parasites and higher trophic positions. It is currently difficult to parse out whether or not smaller, more basal organisms are simply undersampled or actually have fewer parasites, but it is evident that biologists find fewer parasites in these taxa.

## 3 Implications of zeros

A critical question that must be answered before proceeding is; what real effect might these zeros have on our interpretation of parasites in food webs (and ecological communities)? Moreover, does the admission that many of these zeros are methodological artifacts harm our interpretation of existing work? There are two good reasons why we should not be worried about the validity of the patterns thus far observed in parasite-host food webs. First is the curious tendency for certain free-living species to act as hosts for a disproportionate number of parasites in a system (e.g. Rossiter and Sukhdeo, 2011; Anderson and Sukhdeo, 2011). This is the case for every existing parasite-inclusive food web, and partially explains the massive variation in the per-species parasite fauna seen in our meta-analyses. For example, the magnitudes of the observed mean and standard deviation for per-species parasite fauna reported in Poulin (1997) are nearly the same, suggesting that some free-living taxa are heavily burdened by many parasite species, while others act as hosts for very few (if any) parasites. Thus, at least given the data that we have in hand, it may be fair to assume that the addition of parasites by more extensive sampling might only reinforce the observed patterns (i.e. those with many parasites will get more, and those with few will continue to have relatively few). This idea is supported by the observation that many trophically transmitted life cycles settle in the same predator-prey interactions (i.e. an explanation based on food web topology; e.g. Chen et al., 2008; Amundsen et al., 2009; Anderson and Sukhdeo, 2011). If true, the basic patterns and theoretical assumptions about the establishment, persistence and energy flows of parasites will remain largely the same. The issue would simply be one of magnitude, and not pattern.

The second important item is to remember that parasites have come up absent in our surveys for a reason (even after acknowledging biases in the data). Those parasites yet to be identified are likely increasingly rare. Even a fairly coarse survey will successfully capture the more abundant and prevalent parasite taxa in a system, and these are often the ones that are most influential in the patterns and processes under consideration. For example, the eel nematode *Anguillicoloides crassus* is currently considered the most noisome and devastating fish parasite on the planet. In the fifteen years since its introduction, it has come to infect >80% of eels collected along New Jersey’s shores, and the intensity of infection has tripled (personal communication, Mark Sullivan). Even a small sampling of these fish would yield this parasite in our surveys, and it is safe to say that our assessment of it in the context of ecological communities would find it to be much more significant than the aforementioned *L. setiferoides*, which is exceptionally rare. Thus, when we consider the effects of parasitism at the community level (in either the basic or applied sciences), we may have already identified the most consequential taxa. For the purposes of studies interested in the direct impacts of parasites on host dynamics, species-species interaction strengths, or patterns of biomass distribution in communities, rarity might be tantamount to absence. As one example, consider the key finding of Kuris and colleagues (2008), in which parasites were shown to have cumulative biomasss values comparable to those of top predators. This finding was driven almost entirely by the biomass production of trematodes (namely larvae), which was more than an order of magnitude larger than any other parasite group. The addition of any rare taxa found in more extensive sampling would have almost no bearing on the central thesis of the study. Likewise, if the host is itself too rare to thoroughly sample, with the exception of conservation efforts to rescue it from extinction, the host (and its parasites) may be of secondary importance to the dynamics or structure of the systems we seek to understand (called the “commonness-dominance paradigm”, Gaston, 2012). This is by no means a settled discussion in ecology however, as some species that are numerically rare (parasitic or otherwise) can have dramatic effects on the structure and function of the larger community (Lefèvre et al., 2009). For example, metecercariae of *Curtuteria australis* modify the burying behavior of the New Zealandcockle (*Austrovenus stutchburyi*), which, when remaining on the benthic surface, gives rise to an entire community that would not exist in the absence of the parasite (Leung and Poulin, 2007). While this parasite is not rare, its impacts on the system are not in proportion to its numerical abundance.

While there may be some evidence in favor of keeping our current theories intact, not all zeros are inconsequential. For example, when the absences are due to intentional avoidance of parasite groups, our interpretation of the dynamics of these systems may be at risk. Though it’s an extreme example, consider any study of the human population. To be sure, helminth parasites abound in some locations, but we would be remiss if we concluded that viral, bacterial, protozoan or even blood-borne parasites were not important. Yet, they are often ignored in our studies of ecological communities. In truth, though they are not difficult to survey, we have little sense of the relative magnitudes of the community-level effects of different types of parasites (viral, bacterial, helminth and so on), and would likely benefit from an increased effort in that particular arena of ecological parasitology. Further, we remain mere neophytes with respect to the diversity and dynamics of these parasites in non-model organisms (which make up the bulk of ecological communities).

Likewise, the richness of both parasite taxa and life-cycle strategies is rather important. Even if a complete and accurate census of the parasites of a particular system only reinforced the patterns seen in more coarse surveys, the magnitude might be telling. For example, though many have examined the consequences of “environmental perturbation” on parasite-host patterns (e.g. Hernandez et al., 2007; King et al., 2007; Blanar et al., 2011), general patterns remain unclear. This is largely due to the fact that, within the broader field of ecology, “perturbation” is a slippery term (i.e. not all sources of environmental impacts are the same; Rykiel, 1985). But, as has been mentioned elsewhere (Marcogliese, 2005), parasites may be one of the best biological indicators for distinguishing between, and quantifying sources of perturbation. There is still a need to look at the same (or similar) system(s) across gradients of disturbance, using parasite-based metrics. How does a community “shed” parasite life cycles as it is deconstructed? Which types of perturbations are parasites most sensitive to, and can the absence, presence or richness of those parasite life cycles tell us something about the larger community? These are precisely the types of questions that can be addressed with highly resolved food webs.

## 4 Conclusion

While we are perhaps still in the speculative phases of understanding, it appears that few (though non-negligible) zeros are biologically “real,” and, depending on the type of question being asked, the artifactual zeros may or may not matter. Specifically, if we are simply looking to describe the patterns of parasitism in natural communities (or even their causative mechanisms), zeros may not be overly problematic to our interpretations. For example, both Chen and colleagues (2008) and Rossiter and Sukhdeo (2011) evaluated several food webs that differ in how exhaustively parasites were surveyed, and yet found similar overarching patterns in the position of parasites in those food webs. Similarly, it is likely that missing parasites for reasons of rarity (for the host, the parasite or both) may not be of great consequence when trying to work out the dynamics of a system (again, exceptions noted). As a parallel, most free-living groups have been more robustly examined in ecological communities, and the deficiencies in resolution typically do not hinder us from being able to assess their importance (microbial communities not withstanding). The lack of resolution does, however, limit our ability to use poorly fleshed out groups to detect environmental change (almost all of our bioassessments are explicitly built around highly resolved taxonomy; e.g. Karr and Dudley, 1981). Likewise, only food webs with highly resolved parasite groups will allow us to determine if the sources of zeros are meaningful in the context of the state or condition of a system, or whether or not certain types of parasites are more susceptible to perturbation than others. It would be interesting to use either existing long-term surveys or establishing new research programs to track the rate at which new parasite taxa are added with respect to effort and time, so that we might estimate the “true” parasite richness of these systems. For each free-living member of the community, we might record their relative abundance and variation, while at the same time tracking their associated parasites with respect to the number of putative hosts sampled. Just as we use rarefaction curves to approximate the total free-living diversity of a given system (Gotelli and Colwell, 2001), the total parasite community could be estimated from accumulation curves specific to each host survey. Because some of the existing food webs are both highly resolved and represent long-term research programs, retrofitting such estimations may simply be a matter of math, and not more intensive labor. Such methods might satisfy our curiosities with respect to the true parasite:host ratios in nature (see Dobson et al., 2008).

An alternative strategy is to return to studying the dynamics of host–parasite focal species, where the devils so often associated with the details can be brought to light (e.g. Lettini and Sukhdeo, 2010; Bernot, 2013). Perhaps we can learn more about the role(s) of parasitism in ecological communities (and food webs) by knowing a few host–parasite modules well, as opposed to having a superficial or cursory knowledge of the whole. Deconstructing communities into interactions modules or “focal species” partitions (i.e. the host, parasite, and associated species) might allow us to assess the real dynamics of a system in pieces more amenable to intensive surveying and experimental manipulation. This has been the research path of “parasite ecology” in the past, and has been identified as an important and worthwhile endeavor today (Sukhdeo, 2012). While it is true that many of these interaction modules may prove to be of minor relevance in the context of the greater community (though we should not forget that the greater community may still prove relevant to the module), such studies have recently provided evidence for large-scale effects on communities (e.g. Bernot and Lamberti, 2008; Sato et al., 2012). Going forward, perhaps the best validation of our current understanding of parasitism in food webs will come from the union of empirical studies at the population and community levels, in which those details captured in parasite-host studies begin to make sense at larger scales. Without diminishing the importance of findings in existing literature, the reasons behind the zeros observed in food webs should at least caution us to carefully consider and appreciate the possible limitations and caveats in our interpretations of natural systems.

## Figures and Tables

**Table 1:** General description of the sources of “zeros” in food webs containing parasites, where I discuss them, whether the zeros are real or artifact, and the relative frequency in which each type of zero is observed. Frequency categories are: very common, common, less common, and rare, where very common sources are present in nearly all studies and rare sources represent anomalous observations.

Source of zero		Section	Type	Relative frequency
Numerical biases	Undersampled or ignored groups	2.1	Artifact	Very common
	Differences in taxonomic resolution	2.1	Artifact	Very common
	Host or parasite abundance or rarity	2.1	Artifact	Common
	Absence or limits on destructive sampling of hosts	2.1	Artifact	Very common
	Intent or goal of the study	2.1	Artifact	Very common

Spatial or temporal	Spatial scale or system size	2.2	Valid	Common
	Cumulative vs real-time surveying	2.2	Artifact	Common
	Duration of study	2.2	Artifact	Common

Ecological	Parasite life history	2.2, 2.3	Valid	Common-rare^a^
	Host local and geographical distribution	2.2	Valid	Less common-rare
	Environmental constraints or perturbation	2.2	Valid	Less common^b^
	Community (or food web) structure	2.2, 2.4	Valid	Very common

a Commonly, broad host spectra, along with multi-host life cycles, allow many free-living species to serve as hosts even when there are few parasite species. More rarely, the absence of one host in the life cycle prevents the establishment of a parasite in other would-be hosts in a study system.

b Because constraints and perturbations are so diverse, it is difficult to estimate how frequently they limit parasitism in communities.
